# Supplementary material for: Are carbon emissions trading and green financial instruments synergistic? -Comprehensive quantitative research based on content analysis
Source: PLoS One. 2024 Mar 7;19(3):e0298601. doi: 10.1371/journal.pone.0298601 (PMC10919601; doi:10.1371/journal.pone.0298601)
Supplement: S1 Data — (ZIP) [file pone.0298601.s001.zip › Data/地级市Z_F工作报告.zip!/╔╜╢1⁄2/╘μ╫»╩╨/└┤╫╘╣1⁄2╓┌║┼í╛┬φ┐╦╩2╛▌═°í┐_2019.docx]

各位代表：

　　现在，我代表市人民政府向大会报告工作，请予审议，并请市政协各位委员提出意见。

　　一、2018年工作回顾

　　一年来，在省委、省政府和市委的坚强领导下，全市上下认真践行习近平新时代中国特色社会主义思想，深入推进供给侧结构性改革，加快实施新旧动能转换重大工程，抢抓机遇、迎难而上、砥砺奋进，迈出了高质量发展的新步伐。预计全市生产总值完成2400亿元，增长4.5%；一般公共预算收入完成146.7亿元，增长1%，可用财力增长5%；社会消费品零售总额950亿元，增长8.6%；城镇、农村居民人均可支配收入分别增长6.8%和8.3%，经济社会发展总体平稳向好。

　　（一）新旧动能转换全面起势。市级98个重点项目完成投资240亿元，一批重点项目投产运营；成立五个一线指挥部，扎实推进新旧动能转换重大工程项目建设。实施领导干部帮包帮扶“633”工程，高端装备、高端化工等六大特色优势产业主营业务收入增长11%；大数据产业相关企业发展到近200家，被命名为省级大数据产业集聚区；鲁南高科技化工园区等园区通过省级认定。鲁南产学研协同创新示范区加速建设，国家可持续发展议程创新示范区创建跻身候选城市行列；新增院士工作站、工程技术研究中心、企业技术中心等各类创新平台181个，90%的规模以上工业企业建立了产学研合作关系；高新技术产业产值占比达到27%。设立总规模100亿元的市级新旧动能转换基金，发起省内首支规模30亿元的新旧动能转换跨境创投类母基金。35家企业完成股改，丰源轮胎、益康药业在“新三板”挂牌。新增规模以上服务业企业和限额以上批零住餐企业117家，电商交易额突破800亿元，旅游消费总额增长12.7%，现代服务业增加值占服务业增加值比重达到44%。统筹推进农业农村“三区”共建，新发展市级以上新型农业经营主体133家、农业精品园区40家、美丽生态养殖场25个，新增“三品一标”70个，市级以上“一村一品”示范村镇达到52个。

　　（二）改革开放深度拓展。市深改组审议改革方案32个，新争取国家、省级改革试点18项，按时间节点落实改革任务241项。供给侧结构性改革持续深化，“三去一降一补”成效显著，全市过剩产能化解率达到80%以上，减税降费30多亿元，新申报马德里国际商标22件，新增名牌产品、驰名商标9件。国企国资改革取得新进展，组建产业集团或集团化一级企业7个。财税改革不断深化，国地税机构合并顺利完成，全市税收收入完成107亿元，“四税”占比提高5.6个百分点，财政收入质量明显提升。农村改革扎实推进，63%的村完成集体产权制度改革，山亭区列入国家农村综合性改革试点试验区。“双招双引”力度持续加大，成功举办首届枣庄儒商大会、北京招商周等招商活动，新开工外来投资亿元以上项目164个、10亿元以上项目10个；扎实开展“高校行”“城市行”等招才引智活动，在英国成立我市首家海外孵化器，在北京中关村设立首家离岸孵化器，柔性引进各类高层次人才116人。对外开放稳步扩大，外贸进出口104亿元，增长3%。加大园区“腾笼换鸟”力度，盘活土地1976亩。

　　（三）重点攻坚任务取得阶段性成果。深入推进中央和省环保督察反馈问题整改，整治“散乱污”企业762家，PM2.5、PM10浓度分别改善9.7%、7.5%；全面实行河长制、湖长制，全市6个国控河流断面水质均值达到Ⅲ类标准，代表山东省迎接国家河长制中期评估核查，成绩名列前茅；发布《枣庄市山体保护名录》，治理修复破损山体1300余亩，新增造林9万亩。打响脱贫攻坚战三年来，累计实施产业扶贫项目1176个，减贫7.98万人，基本完成脱贫任务，进入巩固提升阶段；加强与威海市、重庆丰都县的扶贫协作，全省扶贫协作现场推进会在我市召开。全力排查化解金融风险，严厉打击非法集资等行为；坚持政、企、银、监协调联动，加强与枣矿集团战略合作，枣庄银行不良贷款得到妥善化解，步入稳健发展轨道；规范政府融资举债行为，政府债务率稳定在合理水平。

　　（四）城乡发展同步提质。“四城同创”扎实推进，国家卫生城市创建顺利通过国家暗访和技术评估，三年来各级财政累计投入21亿元，城市面貌发生巨大变化，群众获得感、满意度大幅提升；国家节水型城市创建完成第一阶段任务；国家生态园林城市、全国文明城市创建全面展开。启动“一河一路”规划建设，基本完成蟠龙河片区概念规划、世纪大道规划设计方案；提前完成城市违法建设治理三年行动任务，累计拆除、整改存量违法建设262万平方米；新建改造城市道路57条，建成城市综合管廊12.4公里，新增建成区绿化面积253万平方米、停车位2.2万个，新增城市集中供热面积228万平方米；智慧城市建设取得新进展，工业、社保、城管等8朵“智慧云”上线运行。编制完成全市乡村振兴战略规划，开展农村人居环境整治三年行动，启动实施镇域路域环境综合整治，新建省级美丽乡村83个，改造农村危房2284户、旱厕11.6万户；新建改造“四好农村路”632公里，新增“户户通”村居1006个。重大基础设施建设加快推进，庄里水库累计完成投资36亿元，大坝主体工程基本完工，移民迁建基本完成；枣庄机场项目预可研报告通过国家民航局评估；快速公交B6线建成通车，枣木高速东延、枣菏高速、万年闸复线船闸等工程进展顺利。

　　（五）民生保障进一步加强。20件惠民实事全面完成年度目标，民生支出占比提高到74.5%。新增城镇就业4.4万人，农村劳动力转移就业5.2万人。城乡低保、特困人员救助等社会保障新标准全面落实。开工建设棚改安置房2.9万套，改造老旧小区72个。完成省定“超大班额”化解任务，投入5亿元，新建改扩建学校14所17万平方米；完成“全面改薄”校舍建设类项目85个，新增学前教育学位9240个。公立医院综合改革稳步实施，分级诊疗扎实推进，医联体总数达到22个。新时代文明实践中心试点工作有序开展。圆满组织省第七届文博会枣庄分会场活动、市第三届群众文化艺术节，完成“枣庄八景”征集评选。成功举办2018枣庄国际马拉松比赛、市第八届全民健身运动会，18届亚运会获得3枚金牌、创历史最好成绩。深入开展“大快严”集中行动、百日安全专项整治，全市安全生产形势持续稳定。省级食品安全市创建全域推进。双拥共建扎实开展。民族宗教、应急管理、外事侨务、妇女儿童、援疆、老龄、残联、档案、史志、物价、人防、地震、气象、红十字等工作取得新进展。

　　（六）发展环境更趋优化。深入贯彻全面从严治党要求，严格落实中央八项规定精神，持续纠治“四风”，坚决做好中央和省委巡视反馈问题整改。深入推进“大学习、大调研、大改进”，扎实开展干部作风建设“九项行动”，集中整治形式主义、官僚主义，政府作风持续改善。办结市人大代表建议66件、市政协提案368件。依法办理行政复议案件160余件，市长热线办复率99%。深化法治枣庄、平安枣庄建设，深入开展扫黑除恶专项斗争，打掉涉黑组织3个，破获涉黑涉恶类案件665件，查处党员干部、公职人员涉黑涉恶和“保护伞”问题27起37人。发布失信黑名单23批次，联合惩戒失信主体1283个。深化“放管服”改革，削减市级行政权力事项1072项，削减幅度达22%；推行“一次办好”集中审批和“减证便民”服务，取消市级证明事项26项，市级行政许可事项全程网办和在线申办率达99.2%；新登记市场主体5万户，增长18.7%。市县机构改革、事业单位分类改革稳妥有序推进，从市、区（市）两级分类改革、职能弱化的事业单位调剂750名编制充实到镇街，形成了关心关爱基层干部的鲜明导向。

　　各位代表！这些成绩的取得，离不开习近平新时代中国特色社会主义思想的科学指引，离不开省委、省政府和市委的正确领导，离不开人大、政协和社会各界的监督支持，是全市人民众志成城、奋力拼搏的结果。在此，我代表市人民政府，向奋斗在各行各业的广大干部群众，向解放军、武警驻枣官兵和中央、省驻枣单位，向在我市创业发展的投资者、建设者，向所有关心、支持枣庄发展的社会各界朋友，表示衷心的感谢和崇高的敬意！

　　我们也清醒地认识到，当前我市发展不平衡不充分的问题突出，传统产业占比较大，产业结构调整、转型升级任务艰巨繁重；科技创新基础不强，新兴业态、高新技术产业总量偏小；支撑区域经济社会高质量发展的大项目、好项目偏少，固定资产投资增长势头减弱；经济外向度偏低，园区发展内生动力不强；城市基础设施欠账较大，公共服务供给能力不足；巩固脱贫成果还需要持续加力，乡村振兴、环境保护、安全生产还有很多薄弱环节，民生短板亟待补齐；营商环境仍需大力改善，中小微企业融资难、成本高等问题比较普遍；少数政府工作人员服务意识不强、担当精神不足，不作为、慢作为、假作为的现象依然存在。对于这些问题，我们将认真分析研究，制定针对性措施，切实加以解决。

　　二、2019年工作安排

　　2019年是新中国成立70周年，是全面建成小康社会的关键之年，也是我市创新转型实现高质量发展的重要一年。我们面临许多可以抓住用好的重大机遇。随着新旧动能转换接续发力，一些新的经济增长点正在加速形成，高质量发展的道路会越走越宽；随着新技术革命的来临，新一轮产业变革正在蓬勃兴起；随着区域协同发展全面提速，我市与周边地区的交通、产业、市场融合度越来越高，合作空间越来越大；随着内需市场持续扩大，新型城镇化、消费升级、补齐民生短板等将全面激活增长潜能，对投资和消费起到双重拉动作用。前不久召开的中央和全省经济工作会议为今年经济工作把向定调，更加坚定了我们凝神聚力抓发展、矢志不渝谋跨越的信心和决心。

　　今年政府工作的总体要求是，以习近平新时代中国特色社会主义思想为指导，全面贯彻党的十九大和十九届二中、三中全会精神，认真落实习近平总书记视察山东重要讲话、重要指示批示精神，科学把握重要战略机遇期新内涵，在省委、省政府和市委的坚强领导下，坚持稳中求进工作总基调，坚持新发展理念，坚持推动高质量发展，坚持以供给侧结构性改革为主线，坚持深化市场化改革、扩大高水平开放，继续打好三大攻坚战，以新旧动能转换重大工程为引领，着力巩固“稳”的基础，挖掘更多“进”的潜力，聚焦创新、决战产业、主攻项目、突破园区、融合城乡、普惠民生，全力加快资源型城市创新转型高质量发展。

　　今年全市经济社会发展的主要预期目标是：生产总值增长5—6%；一般公共预算收入增长3%左右；外贸进出口稳定增长；城镇、农村居民人均可支配收入分别增长6.5%和7.5%左右；城镇登记失业率控制在3.5%以内；全面完成省下达的年度节能减排降碳约束性目标。重点抓好以下七个方面的工作：

　　（一）坚定不移推动产业升级，举全市之力振兴实体经济

　　加快制造业强市步伐。坚持以“四新”促“四化”，开展制造业提质增效行动，大力发展高端、智能、绿色制造，重点培优培强六大特色优势产业。通过三到五年的努力，力争制造业规模突破5000亿元。高端装备产业，抓好高端机床研发及产业化、智能装备、智能制造产业园等项目，着力突破关键技术与核心部件，实现枣庄制造业的加速蝶变。高端化工产业，依托省级化工园区，重点推进兖矿鲁南化工高端改性聚甲醛与己内酰胺、联泓新材料研发转化基地等项目，延伸煤气化，提升煤焦化，发展精细化工，加快千亿级化工新材料板块强势崛起。大数据产业，以鲁南大数据中心、滕州大数据产业园为载体，建设云存储灾备基地和大数据清洗加工基地，积极创建国家大数据综合试验区；启动科云大厦项目建设，推进鲁南大数据中心扩容；加快建设汉芯半导体产业园等项目，延展大数据关联业态；继续办好数字中国（枣庄）峰会；深入推进智慧枣庄“16161”工程，编制实施大数据应用行动方案，让数据创造更多经济价值、社会价值。锂电产业，重点推进中材锂膜二期、丰元锂能高镍电池材料、振兴科技负极材料、航天科工新能源军民融合产业园、华亿比科动力电池扩建等项目，打造全产业链发展格局。光纤产业，依托东方光源集团有限公司，重点推进光纤棒、光纤拉丝等项目，加快向光棒、光纤、光缆、光元器件制造一体化转型升级。医药产业，重点推进海王医疗器械产业园、华润三九中成药数字化车间、中国药科大学制药公司原料药厂等项目建设，进一步做大医药产业集群。继续实施高端、高水平技改三年行动计划，重点抓好50个工业和信息化项目，推动煤电、建材、纺织、造纸、食品等传统产业转型升级。加强资源整合、政策集成，促进军民融合产业实现突破发展。

　　做优现代服务业。推进“文化+旅游”，依托台儿庄古城旅游集团有限公司，整合全市优质旅游资源，做大资产规模，力争实现台儿庄古城旅游集团有限公司上市效益最大化。围绕山水生态、运河风情、工业体验、圣贤寻踪、乡村民俗等文化旅游资源，高标准策划全域旅游线路。开展媒体枣庄行系列活动，展现枣庄风情，讲好枣庄故事。积极融入大运河文化带建设，抓好台儿庄文化产业园、墨子文化城等项目，推动大运河从“地理空间”迈向“文化空间”。推进“医疗+康养”，抓好市医养康复中心等项目建设，发挥市立医院等公立医院的辐射带动作用，引导民间资本参与康养机构建设，打造区域性医养融合发展高地。推进“物流+商贸”，加快枣庄铁路物流园、滕州新港等项目建设，推动公路、铁路和临港物流园运输线路无缝对接，打造公铁水一体化的现代物流体系。鼓励各类商贸市场与大型物流企业联合发展。着力繁荣以万达、银座、贵诚为中心的城市商圈，改造提升一批商业街区。支持骨干商贸物流企业开展标准化、信息化建设，积极创建物流标准化试点市。

　　做大龙头骨干企业。深入实施领导干部帮包帮扶“633”工程，力争主营业务收入过50亿元企业达到5家，过20亿元企业达到10家。支持枣矿集团发展本部经济，通过内配外调实现由生产煤炭向经营煤炭转变，着力抓好铁水联运、智能集装箱加工制造基地、智能康养高端设备生产基地、智慧快递中心园区、再生资源冶炼深加工循环利用产业基地、钢铁智慧物流仓储加工中心等项目建设，加快产业转型和新动能培育，尽快实现营业收入、资产规模双双突破1000亿元。支持枣矿集团依托枣庄银行，打造现代交易银行模式。积极培育“专精特新”小巨人企业。建立股改上市企业动态资源库，完成40家企业股改，着力推进联泓新材料、泰和水处理等公司上市。加强企业家队伍建设，激励引导企业家干事创业。

　　（二）坚定不移推动创新发展，激发新旧动能转换的强大动力

　　加快推进“一城两区”建设。积极创建国家创新型城市，进一步增强科技创新综合实力。鼓励支持企业建立研发平台，加大全社会研发经费投入。强力推进鲁南产学研协同创新示范区建设，加快建设枣庄学院新校区、枣庄学院科教产业园、北中医枣庄医院二期、互联网小镇等项目，促进创新资源要素整合集成。积极创建国家可持续发展议程创新示范区，推动科技创新与社会发展、运河文化与区域经济深度融合。

　　突出抓好创新平台建设。加快中科院化工新材料产业化基地、中国建筑材料科学研究总院山东分院、常州大学国家级技术转移中心枣庄分中心、浙江大学山东工研院等平台建设，探索组建产业创新联盟，打造“政产学研金服用”创新共同体。依托国家级高端制造装备协同中心、北航机床创新研究院、北理工鲁南研究院，建设精密机床协同创新中心。鼓励企业与高校、科研院所共建工程技术研究中心、重点实验室、院士工作站、科技孵化器，攻克一批制约产业发展的“卡脖子”技术问题。年内，新增各类创新平台50个以上。实施政、研、企合作“彩虹工程”，举办高层次科研成果推介会，推动科研机构与企业务实合作。加强知识产权保护，培育一批科技成果转移转化服务机构，促进技术成果转化应用。

　　着力营造创新创业创造的浓厚氛围。全面落实科技创新扶持政策，引导企业加大研发投入，承担更多科技计划项目，培育科技型中小企业50家，新增高新技术企业15家。实施标准引领工程，鼓励企业参与国家标准、行业标准制定，争取更多标准化技术组织落户枣庄。围绕“增品种、提品质、创品牌”，深入开展高质量发明创造攻坚行动，积极争创中国驰名商标、山东名牌产品、省长质量奖，鼓励企业增加有效商标注册量。建设运营好创业大学、创业学院，打造一批省、市级创业示范园区，扶持一批创业孵化基地、众创空间、创业“梦工厂”。探索股权激励、兼职取酬、科研成果收益分红机制，用活用好创业投资引导基金、创业补贴。发挥好枣庄学院、枣庄职业学院等高校作用，培养更多实用型创业创新人才。大力弘扬工匠精神、劳模精神，深入实施高技能人才提升工程，加快培养一批能工巧匠和技术革新能手。设立“墨子创新奖”“鲁班工艺奖”，形成激励创新创造的鲜明导向。

　　（三）坚定不移推动改革开放，增强经济发展活力

　　凝心聚力抓好重点领域和关键环节的改革。深化金融改革，完善引进金融机构的财税激励政策，调动各区（市）引进银行、保险、证券机构的积极性。加快国家级普惠金融改革试验区建设，探索建立凤鸣基金小镇，引导更多资金流向“三农”和小微企业。深化财税改革，完善现行财政体制，增强市级调控能力，支持县域经济做大做强。加快构建事权与支出责任相适应的财政体制，将更多的财力向基层倾斜。推进税收征管体制改革，稳妥有序划转社会保险费用和非税收入征管职能。深化投融资改革，做大做强市基础设施投资公司、市土地发展公司，为全市重大基础设施建设提供强力支撑；规范市财金集团运营，发挥政府股权投资引导基金和新旧动能转换基金的撬动作用，加快建立各类产业投资基金，构建跨境产业金融服务体系，促进基金与产业深度融合发展。新设立、组建若干国有投资控股公司，优化国有资本配置，增强国有资本控股企业的投资、融资能力，更好地支持全市重大项目建设。深化国企国资改革，实施国有企业战略性重组，组建运输集团、水务集团、开发建设集团等市管企业。加快国有资本投资运营公司改组、改建，积极推进国有资本投资运营公司试点。支持市属国有企业与各类国有资本、集体资本、非公有资本等交叉持股、相互融合，加快股权结构优化，积极发展混合所有制经济。深化“一次办好”改革，按照“放无可放、减无可减”要求，进一步推动行政权力事项下放区（市）、镇街。发挥好市、区（市）行政审批服务机构作用，推动市级审批关口前移，构建一体化、扁平化的审批体制。深化“互联网+政务服务”，实现政务服务事项数据全打通、全归集、全共享、全对接。继续优化办理流程，扩大“一链办理”“一网通办”事项范围，促进政务服务更加高效便捷。上半年完成市民服务中心建设，市直所有审批事项全部进驻。

　　凝心聚力抓好“双招双引”。强化企业招商、以商招商，形成“引来一个、带来一批”的集聚效应。强化产业招商，围绕特色优势产业建链、补链、强链，引进一批上下游配套项目，全面提升产业规模和竞争力。强化“项目+基金”招商，设立若干产业投资基金，实行市场化、专业化管理运作，真正用基金吸引项目、优选项目、扶持项目。强化专业招商，选配具备专业能力、专业精神的招商队伍，聘请一批招商大使、招商专员、招商顾问，提高招商成功率。盯紧世界、国内、民企“三个500强”，深化与中国建材集团、国科控股、枣矿集团、兖矿集团等央企、省企的合作，集中力量招引一批大项目、好项目。精心组织北京、长三角、珠三角、欧美、日韩、香港等境内外招商活动。发挥各地枣庄商会作用，推动“枣商回归”。完善“双招双引”优惠政策，建立要素资源跟着项目走的保障机制，发挥考核激励导向作用。继续实施柔性引才“百人计划”，开展高层次人才枣庄行、“枣庄—高校人才直通车”活动，新引进“万人计划”、泰山系列等高端人才100人以上。

　　凝心聚力抓好园区改革创新发展。统筹推进园区管理体制机制创新，实行“区辖镇街”模式，赋予开发区本级政府经济管理权限和部分行政权限。对急需的高层次人才和“双招双引”人才实行雇员制管理，建立绩效考核、绩效薪酬制度。推行“亩均投资效益”评价，对开发区实行分档激励，对企业实行差别化要素扶持。加快“腾笼换鸟”，着力处置“僵尸企业”。加大清理处置低效利用土地和闲置土地力度，腾出更多空间用于高质量项目。统筹开发区产业定位布局，突出主导产业培育，大力发展“飞地”经济，建立“飞地”项目税收利益分享机制，对市内各级政府引进的跨区域投资产业项目，打破行政区域界线，实行项目转出地（招商地）与转入地（承接地）主体税收共享。完善园区设施配套，积极建设智慧园区，增强综合承载能力。支持枣庄高新区规划布局新的产业聚集区，鼓励高新区、滕州市和有关区制定特殊政策，设立“企业发展特别扶持资金”，对企业发展战略研究、技术创新、产品研发、市场开拓、员工培训、职工福利等实行无偿资金扶持，吸引各类企业在枣庄设立总部或区域总部，打造“枣庄总部经济群”。

　　凝心聚力抓好对外贸易。深入开展“双唤行动”“双培工程”，继续做大外贸综合服务平台、跨境电商平台，支持建设跨境电商产业园，提高出口便利化水平。巩固既有优势出口产品的出口份额，分类推动外贸转型示范基地、外贸出口基地建设。支持区（市）创建省级服务贸易创新发展试点，积极培育领军企业、知名品牌和特色服务出口基地。推进海关特殊监管区建设，拓展青岛港枣庄内陆港区服务功能，实现进出口贸易“铁海直通直入”。加大与中欧班列对接力度，更好地开拓“一带一路”沿线新兴市场。鼓励外资企业增资发展和并购投资，扩大利用外资规模。

　　凝心聚力抓好民营经济发展。全面贯彻省“民营经济35条”“非公有制经济10条”，进一步简政、减税、降费，让民营企业更有获得感。开展“一企一策”专家入企服务活动，帮助企业解决融资、用地等难题。实施小微企业“双升”战略，推进“个转企、小升规、下转上”。深入开展优化企业开办、工程建设项目审批等10大专项行动，打造更加优质高效的营商环境。建立“政商直通车”平台和企业紧急事态应对机制，做到“企业需要时，政府无处不在；企业不需要时，政府无声无息”。

　　（四）坚定不移推动新型城镇化提质扩容，打造美丽家园

　　着力推进区域融合发展。完善我市组团城市发展战略规划，优化组团城市空间布局，强化新城区的城市主中心地位。加快新城区与市中区融合发展，重点推进世纪大道建设、蟠龙河两岸两条堤顶快速路建设、枣曹线城市道路升级、齐陶路西延、光明大道提升，实现新城与老城的快速连接，促进人流、物流、资金流加速流动和产业融合。加快新城区与滕州市融合发展，积极探索试行滕州市与新城区之间高速公路免费通行，启动祁连山路北延、荆泉路（或龙泉路）南延规划建设，做好B6线配套工程建设和管护提升工作。加快新城区与薛城区一体化深度融合发展，理顺城市建设管理体制机制，统筹新城区、薛城区的空间规划、产业布局、公共服务和文体商业设施，完善新城区南部路网，推进京台高速枣庄段（城区段高架）改扩建。发挥教育、医疗、交通优势，促进薛城微山同城化发展。推动跨市域协同发展，抢抓国家实施淮河生态经济带建设、淮海经济区协同发展和省里支持鲁西崛起的机遇，加强与周边地区的规划衔接和项目对接，积极推动徐枣城铁、台儿庄S234接徐州S252、S241临徐线与江苏S270连接线、台睢高速公路、观音机场台儿庄城市候机楼等互联互通项目规划建设；深化与周边城市经贸、能源、金融、人文合作，推动旅游市场共建共享、产业协同发展。

　　着力推进“一河一路”规划建设。以蟠龙河综合整治和世纪大道规划建设为带动，推进新城区“北跨、南拓、东融、西进”。按照“高质量生态建设示范区”的定位，实施蟠龙河流域起步区综合整治，开工建设蟠龙河流域各类水利工程项目，开工建设沿蟠龙河两岸两条堤顶快速城市道路。围绕蟠龙河流域，植入文化体验、休闲旅游、度假康养等元素，沿河布局产业功能区，着力打造高颜值的生态画廊、高品位的文化长廊、高效益的经济走廊。开工建设世纪大道，实施民生南路、祁连山南路等道路建设，构建中心城区快速路网体系。

　　着力推进城市有机更新。持续开展“四城同创”，深入实施城市品质提升三年行动，完善基础设施功能，补齐城市发展短板。用好国家棚改政策，开工建设棚改安置房1.6万套。采取市场化、法治化手段，综合整治“烂尾楼”。改造老旧小区78个。开展既有多层住宅增设电梯试点。新增城市集中供热200万平方米，建设城市地下综合管廊6公里。推动海绵城市连片建设。加快城市公共厕所提档升级，改造涉农街道无害化卫生厕所1万户。持续开展违法建设治理专项行动，深度整治背街小巷，打造精品示范街区。实施道路畅通工程，打通城区路网“微循环”。合理布局停车泊位，支持立体停车场建设。实施运输绿色化改造，积极创建国家公交都市。结合“智慧城市”建设，提高城市管理数字化、精细化水平。

　　着力推进重大功能性基础设施建设。完成枣庄机场项目预可研批复，争取早日开工建设。加快枣菏高速、枣木高速东延、新台高速工程建设。启用万年闸复线船闸。完成庄里水库大坝枢纽工程，达到蓄水条件；加快推进抽水蓄能电站前期工作。完成十里泉电厂运煤专线外迁工程、外电入鲁1000千伏特高压“一站两线”工程。

　　（五）坚定不移推动乡村振兴，写好枣庄特色篇章

　　培育更多的富民产业。加快发展农业“新六产”，实施好国家产业兴村强县示范行动项目，抓好现代田园综合体建设。加大农业龙头企业培育力度，新增市级农业龙头企业20家以上。实施信息进村入户工程、农商互联工程，加快村居“益农信息社”建设，培植一批电商产业园、示范镇村。坚持就地城镇化、镇村一体化、乡村特色化，推动新生小城市、重点示范镇和各类特色小镇提档升级，打造一批休闲小镇、生态小镇、创意小镇。实施“厚道枣庄人、放心农产品”品牌引领行动，进一步提高滕州马铃薯等农业区域品牌知名度，新认定“三品一标”30个以上。引导发展生态养殖，新建美丽生态养殖场20个。支持峄城区高标准编制石榴产业发展专项规划，扩大种植面积，大力发展精深加工和生态旅游，提高产业附加值，打响石榴特色品牌。支持区（市）创建省级以上农产品质量安全县。开展粮食绿色高质高效创建工作，推进全面全程机械化示范镇、示范村建设。

　　营造更美的人居环境。深入开展农村人居环境整治三年行动，大力实施农村“五化”“七改”工程，创建美丽乡村160个。以镇街驻地、两高沿线、国省干道、城际交界处为重点，全面推进镇域路域环境综合整治，坚决消除“脏乱差”。加快“四好农村路”建设，两年内自然村全部通等级公路，实现村居道路“户户通”全覆盖。抓好“三清一改”村庄清洁行动，完善农村“厕所革命”后续管护机制，提升城乡环卫一体化水平。稳步推进农村清洁取暖。加快乡村造林步伐，建成省级森林乡镇2个、森林村居20个。

　　留住更浓的乡土记忆。继续实施乡村记忆工程，保护好古桥、古井、古树、古街巷、古建筑，加强非物质文化遗产保护和传承，打造一批综合性、活态化乡村博物馆，彰显“乡土味道、鲁南风情”。全面铺开新时代文明实践中心建设，以社会主义核心价值观为引领，培育文明乡风、淳朴民风、良好家风，引导群众移风易俗。深入实施乡村儒学推进计划，传承弘扬农耕文化，鼓励支持挖掘乡村文化、编修镇村史志，保持文化遗产的完整性和延续性。

　　释放更强的发展活力。健全城乡融合发展机制，促进土地、资本、人才等要素在城乡间双向流动。全面加快农村集体产权制度改革，开展集体经营性建设用地入市改革。推进“房地一体”登记试点，推动农村不动产进入市场抵押、交易。探索集体资产、农民资产与城市资本合作经营模式，加快盘活农村闲散土地。鼓励村党支部领办合作社，健全村集体收入增长长效机制，全面消除集体经济空壳村。深化供销社综合改革，大力发展农业全程社会化服务。积极推动基层农技人员轮训、科技特派员下乡，激励各类人才在农村广阔天地大显身手。

　　（六）坚定不移推动生态文明建设，夯实绿色发展根基

　　持续打好碧水保卫战。深入实施人工湿地水质净化、河流入库水质保障等工程，全面提升流域水污染治理水平。进一步完善河长制、湖长制组织体系，落实河管员、河道警长，完成全市河湖划界工作。继续开展“清河行动回头看”和“清四乱”，保持境内河流断面水质稳定达标。严格执行工业废水排放标准，确保达标排放。实施城市垃圾处理、污水处理提质增效三年行动，新开工垃圾发电项目2个，新增生活垃圾日处理能力1300吨；大力实施城区雨污分流，提高污水管网覆盖面，新增污水日处理能力7万吨。巩固城区黑臭水体治理成果，逐步消除镇村黑臭水体。深入推进国家节水型城市创建，开展农村饮水安全两年攻坚行动，全面加强饮用水源标准化建设，保障居民饮水安全。

　　持续打好蓝天保卫战。深入开展大气污染防治行动，进一步改善二氧化硫、氮氧化物、PM2.5等指标。突出精准治霾，分类治理施工和道路扬尘、餐饮油烟、机动车船超标排放等问题，重点加强涉气企业综合整治。排查治理“散乱污”企业，改造退出一批玻璃、焦化、砖瓦等重污染企业。积极推广洁净煤，进一步削减煤炭消费总量。继续抓好建筑节能，大力发展绿色建筑和装配式建筑。坚持全年全域秸秆禁烧，推广秸秆综合利用。建立镇街空气环境质量通报、考核奖惩制度。探索建立跨行政区域协作机制，加强重污染天气预报预警，形成横到边、纵到底的协同治污网络。

　　持续打好净土保卫战。开展生态循环农业示范行动，推进国家农业绿色发展试点先行区建设。开展化肥减量使用、农药使用零增长行动，抓好地膜回收资源化利用。加快土地污染状况详查，抓好产业转型腾退土地治理修复。建立危险废物全过程监管体系，严厉打击非法跨界转移、倾倒等行为。

　　持续推进生态修复。加大破损山体修复力度，年内治理破损山体2000亩。严厉打击私挖盗采矿产资源行为，实现保护、开发与修复有机结合。严守生态保护红线，切实管住管好重要生态空间。大力实施退耕还林、退耕还湿、塌陷地治理、水土流失综合整治。抓好“绿满城乡、美丽枣庄”绿化行动，实施绿道提升工程，推进全域绿色生态廊道建设，完成造林面积6万亩。深入开展城市绿荫增补、绿地公园提升、生态街巷创建专项行动，加快创建国家生态园林城市。全面推进“四减四增”，打好八场标志性战役，促进生态环境质量持续改善。

　　（七）坚定不移推动发展成果更多惠及百姓，提升群众幸福感

　　巩固脱贫攻坚成果。围绕“巩固、接续、长效”要求，因户因人施策，细化实化帮扶措施，注重开发式扶贫和保障性扶贫相统筹，兜牢脱贫底线。开展实用技术和劳动技能培训，大力发展产业扶贫基地、扶贫车间、劳务扶贫合作社，加强扶贫项目资产管理，抓好农村存量危房改造。坚持扶贫与“扶志”“扶智”相结合，加大以工代赈力度，引导贫困群众用勤劳双手创造美好生活。强化农村贫困人口动态监测管理，构建稳定脱贫长效机制。

　　完善公共服务体系。统筹县域义务教育一体化改革发展，推动台儿庄、峄城创建全国义务教育优质均衡发展县，持续抓好“大班额”问题化解。全面完成中小学教师“县管校聘”管理改革。实施第三期学前教育三年行动计划，新建、改扩建普惠性幼儿园33所。资助贫困家庭学生5万人次。巩固国家卫生城市创建成果，启动创建第一批全国健康城市。深化医药卫生体制改革，推行分级诊疗、家庭医生签约服务。实施基层医疗卫生能力提升行动，培训全科医生97名。着力增强中医药、产科、儿科服务能力。办好市第九届全民健身运动会。持续开展守护舌尖安全整治行动，加强食品摊点、小餐桌、外卖食品安全监管，积极争创省级食品安全市。合理规划小区非机动车停放区，设置充电桩等便民设施。深化全国文明城市创建，积极开展交通秩序、违规养犬、滥贴广告、物业乱象等专项整治，让文明渗入城市“肌理”、融入百姓生活。

　　繁荣发展文化事业。举办好省第八届文博会枣庄分会场活动、第四届群众文化艺术节、第四届鲁班文化节。抓好公共图书馆、博物馆、文化馆建设，增强公共文化服务供给能力。完成铁道游击队纪念馆建设，开展“讲述红色历史、传唱红色歌曲、展演红色传奇”系列活动。做好红色教育产业规划，丰富铁道游击队、八路军115师、运河支队等载体内涵，创新纪念形式，增强教育效果。深入实施中华优秀传统文化传承发展工程和文艺精品工程，推出一批具有浓郁地方特色的精品力作。

　　进一步提高社会保障水平。深入实施全民参保计划，完善社会保险统收统支机制。加快推进医保支付方式改革，建立病种标准动态调整机制。加强高校毕业生、返乡农民工、退役军人等重点群体就业创业援助工作，开展失业人员技能培训。健全老年人、残疾人、农村留守儿童等关爱服务体系，加强社会救助、社会福利、优抚安置等工作，加快发展社会慈善事业。

　　加强和创新社会治理。深化平安枣庄建设，深入实施网格化管理，加快建设“雪亮工程”，完善立体化治安防控体系。纵深推进扫黑除恶专项斗争，坚决铲除各类黑恶“毒瘤”和“保护伞”。加强地方金融监管体系建设，加大非法金融打击力度，坚决守住不发生系统性、区域性金融风险的底线。强力推进风险隐患排查管控整治，突出抓好煤矿、化工、非煤矿山、道路交通等重点行业领域专项治理，坚决防止重特大安全生产事故发生。坚持依法治市，深入推进以宪法为重点的“七五”普法。完善矛盾纠纷化解体系，保障群众合法利益诉求。探索实施信访积案专家会审评议制度，提升信访事项办理满意度。进一步完善社会信用体系，坚决惩治各类失信“老赖”，全面建设“诚信枣庄”。

　　三、加强政府自身建设

　　改革开放进入新时代，各级政府工作人员要以振兴枣庄为己任，持续转作风、提效能、优环境，全面建设“忠诚干净、担当实干、依法行政、公开透明”的服务型政府。

　　（一）旗帜鲜明讲政治。树牢“四个意识”，坚定“四个自信”，做到“两个维护”，自觉在思想上政治上行动上同以习近平同志为核心的党中央保持高度一致。强化政治责任，保持政治定力，把准政治方向，提高政治能力，坚守对党绝对忠诚的政治品格。始终绷紧政治纪律和政治规矩这根弦，令行禁止、不打折扣，确保党中央、国务院和省委、省政府各项决策部署落到实处。坚定理想信念，强化宗旨意识，不忘初心、牢记使命，把以人民为中心的发展思想贯穿政府工作全过程。持续推动政府系统思想大解放，破除一切不利于发展的桎梏，激活一切有利于创新的因子，引导广大干部坚决闯、大胆试、加油干。

　　（二）规范履职讲法治。严格执行重大行政决策法定程序，切实管好政府“有形之手”，放活市场“无形之手”。依法接受人大法律监督和工作监督，主动接受政协民主监督，自觉接受监察监督、群众监督和舆论监督。充分发挥各类专家智库作用，提高科学决策水平。开展专项行政执法监督检查，认真履行行政复议职责。健全政务公开、新闻发布和网络诉求跟踪办理等制度，提升政务服务热线、政风行风热线办理质量。全面完成市县政府机构改革任务。

　　（三）真抓实干讲担当。围绕2019“工作落实年”，深入推进一线作战抓落实行动，更多地到项目现场去、到困难和问题多的地方去，把各项工作向深处、向细处、向实处推进。坚持一切工作项目化、重点任务清单化，健全工作责任分解机制，加强过程管理和考评问效，确保定下的事干一件成一件。继续精文减会，规范督查考核，控制开展监督检查、索要材料报表的总量和频次，切实为基层松绑减负。鼓励创造性贯彻落实，对真抓实干、成效明显的区（市）、镇街和部门加大正向激励支持力度，形成干事创业、争先进位的良好氛围。健全容错纠错机制，大力褒奖忠于职守、甘于奉献的“老黄牛”干部，敢想敢干、敢闯敢拼的开拓型干部，打出崇尚实干、带动担当、撑腰鼓劲的组合拳。

　　（四）克己奉公讲纪律。坚决扛起全面从严治党主体责任，锲而不舍落实中央八项规定精神。持之以恒纠治“四风”，重点整治形式主义、官僚主义突出问题。把群众意见作为“最好的镜子”，对反映强烈的作风问题开展专项整治，整改不到位不放过、群众不满意不放过。强化审计监督，加强对公共资金、国资国企、公共资源交易等重点领域监管。牢固树立“过紧日子”的思想，严格控制“三公”经费和一般性支出。全市政府工作人员要严以修身立政德、勤勉踏实干事业，树立为民务实清廉的良好形象。

　　各位代表！枣庄跨越追赶的历史重任鞭策着我们，枣庄400多万人民的殷切期盼激励着我们。使命在肩，初心如磐。让我们更加紧密地团结在以习近平同志为核心的党中央周围，高举习近平新时代中国特色社会主义思想伟大旗帜，在省委、省政府和市委的坚强领导下，保持永不懈怠的精神状态和一往无前的奋斗姿态，锐意进取、埋头苦干，加快建设自然生态宜居宜业新枣庄，以优异成绩庆祝中华人民共和国成立70周年！
